# Bean Consumption Accounts for Differences in Body Fat and Waist Circumference: A Cross-Sectional Study of 246 Women

**DOI:** 10.1155/2020/9140907

**Published:** 2020-06-06

**Authors:** Larry A. Tucker

**Affiliations:** College of Life Sciences, Brigham Young University, Provo, UT 84602, USA

## Abstract

Beans and other legumes have multiple nutritional qualities that reduce the risk of many diseases. However, the link between legume intake and obesity remains unclear. Therefore, the present study was designed to examine the association between bean intake, body fat percentage (BF%), and waist circumference, in 246 women. BF% was measured using dual-energy X-ray absorptiometry (DXA). Bean intake was assessed using the Block Food Frequency Questionnaire and indexed using total cups of bean-based food items and also factor scores derived from a factor analysis showing adherence to a bean-based dietary pattern. Bean consumption was expressed as cups per 1000 kilocalories. R\egression results showed that the relationship between bean intake (total cups) and BF% was inverse and linear (*F* = 7.4, *P*=0.0069). Moreover, with bean consumption being divided into tertiles, there were mean differences across groups in BF% (*F* = 7.4, *P*=0.0008) and waist circumference (*F* = 4.2, *P*=0.0164). Specifically, women who consumed moderate or high amounts of beans had less body fat and smaller waists than those with low intakes. Similarly, using tertiles to categorize participants based on adherence to a bean-based dietary pattern, developed using factor analysis, those with low adherence had higher BF% (*F* = 7.9, *P*=0.0005) and larger waists (*F* = 4.5, *P*=0.0118) than their counterparts. The associations remained significant after adjusting for potential confounders. In conclusion, beans and other legumes seem to have dietary qualities that may be beneficial in the battle against obesity.

## 1. Introduction

Pulses are the edible dried seeds of legumes. According to the Food and Agriculture Organization (FAO), there are numerous pulses used as sources of food. One is the common bean (*Phaseolus vulgaris* L.). It is among the leading pulse crops produced in the world. Beans and other legumes have multiple nutritional qualities that help reduce the risk of several diseases [1, 2]. In spite of the many health benefits of eating legumes, current consumption levels are low [3, 4].

Obesity has become one of the most common diseases in developed countries. According to Rebello et al., replacing energy-dense foods with legumes can have favorable effects on obesity [5]. Beans are low in fat and the glycemic index. They are high in fiber and plant protein. They contribute significantly to satiety and they improve the gut microbiome. Clearly, they have the potential to play a meaningful role in the battle against obesity.

Despite the many dietary qualities of beans, actual weight loss results have not always matched expected outcomes. Among four weight loss studies that used negative energy balance designs [6–9], only one resulted in greater weight loss in the legume intake group compared to controls [6]. Nonetheless, when these four investigations were combined into a meta-analysis, weight loss findings were statistically significant in favor of legume consumption compared to controls [10]. Similarly, when a neutral energy balance design was applied, only one of 17 weight loss studies favored legume intake over controls [11]. But when all 17 investigations were combined, meta-analysis supported legume consumption over controls for weight loss [10]. Similarly, in a meta-analysis by Viguiliouk et al., when combined, 21 studies produced more weight loss in the legume group than in controls [2].

To date, only a handful of studies have been designed to investigate the effect of legume intake specifically on body fat. Two studies used negative energy balance designs and neither was statistically significant [6, 9]. Combining them into a meta-analysis did not change the outcome [10]. Four investigations employed a neutral energy balance design and none of them were significant [10]. Combined into a meta-analysis, the results were borderline significant in favor of legume consumption over controls (*P*=0.06) [10].

Overall, the majority of individual investigations studying the relationship between legume intake and body weight and body fat have resulted in nonsignificant findings. As part of a comprehensive literature review, Williams et al. concluded, “There is insufficient evidence to make clear conclusions about the protective effect of legumes on weight” [12]. Failure to find significant evidence could be partially due to the use of small sample sizes and the lack of statistical power. Furthermore, among studies focusing on body fat, measurement error could be a problem, especially for those using bioelectrical impedance [13]. Lastly, although randomized controlled trials are often considered the gold standard design, compliance can be a problem. Some investigations have required participants to eat significant amounts of legumes that subjects were not accustomed to. Therefore, compliance could be an issue, which typically weakens associations.

The present investigation was designed to diminish some of the concerns of other research studies. Specifically, a large sample (*n* = 246) was employed and statistical power was good. Additionally, body fat was assessed using dual-energy X-ray absorptiometry (DXA), a valid and reliable method that produces little measurement error. Also, compliance was not an issue because participants were not required to consume a unique bean-based diet but were asked to report their typical intake of beans and other legumes. Given these adjustments, the primary purpose of the study was to determine the relationship between legume intake, particularly bean consumption, and body fat levels in women. Waist circumference was included as an outcome measure to index abdominal obesity. An ancillary objective was to ascertain the influence of age, education, energy intake, and physical activity on the bean intake and body composition associations.

## 2. Methods

### 2.1. Study Design and Sample

Participants were recruited from more than 20 cities in the Mountain West, USA. Newspaper advertisements, company emails, posters, and flyers were used to contact potential subjects. Participants were delimited to apparently healthy, nonsmoking women because the study's outcomes were body fat percentage and waist circumference. Women and men differ markedly on these two variables [14]. Therefore, if both women and men were included, they would need to be analyzed separately. To maintain the same statistical power and include both men and women, the sample would have to be doubled. Resources were not sufficient to conduct the study with 500 participants. Several well-known investigations have delimited their samples to one sex, such as the Women's Health Study and the Health Professionals Follow-Up Study.

A cross-sectional design was employed. Subjects were primarily non-Hispanic white, employed part time or full time, and married, and about 1/3 reported at least some college credits. Each data collection protocol of the study was reviewed and approved by the university institutional review board (IRB). The study methods were carried out in accordance with the approved protocols. Also, all participants signed an informed consent document approved by the university IRB before any data were collected.

### 2.2. Measures

In the present study, the exposure variable was bean consumption, indexed using two strategies. Body fat percentage (BF%) and waist circumference were the outcome variables. Age, education, energy intake, and objectively measured physical activity level were included as covariates to control for their influence on the relationship between bean intake, body fat percentage, and waist circumference.

#### 2.2.1. Bean Intake

To quantify bean and legume consumption, participants completed the full-length Block Food Frequency Questionnaire (Nutrition Quest, Berkeley, CA), originally developed at the National Cancer Institute under the direction of Gladys Block. The Block Food Frequency Questionnaire is 8 pages in length with items focusing on serving size and frequency of consumption of more than 100 different foods and beverages, including four items specifically about beans and legumes: (1) refried beans or bean burritos; (2) chili with beans (with or without meat); (3) baked beans, pintos, or other dried beans; and (4) bean, split pea, or lentil soup. Each participant was given an additional page containing pictures showing 7 portion sizes on plates and in bowls, so they could precisely record serving sizes. The full-length Block Food Frequency Questionnaire is considered a valid and reliable instrument for assessing dietary intake and has been employed in numerous investigations [15, 16].

For the four questions about consumption of beans and legumes, participants marked one of nine choices about “how often” each of the bean dishes was eaten: never, few/year, once/month, 2‐3 times/month, once/week, twice/week, 3‐4 times/week, 5‐6 times/week, or every day. Subjects also reported “how much” was consumed each time, aided by the page of illustrations showing plates and bowls: 1/4 cup, 1/2 cup, 1 cup, or 2 cups. Consumption of each of the four bean dishes was estimated using the number of cups eaten annually. Total bean intake was calculated by summing the cups reported for each of the four bean-based food items. Because adults who consume more total energy tend to eat more of a particular food, consumption was expressed as cups of legumes consumed per 1000 kilocalories (kcal).

Although the consumption of each of the four bean-based food items was reported in cups, the quantity of beans in each of the bean dishes likely varied. Therefore, an additional method was used to index bean and legume intake. Specifically, a factor analysis was employed using the four bean-based food items to create a single bean “construct” or “factor.” Individual factor scores on the bean construct were used as an alternative bean intake exposure variable.

#### 2.2.2. Body Fat Percentage

Body fat percentage (BF%) was measured using dual-energy X-ray absorptiometry (DXA), with a Hologic QDR 4500W (Hologic, Bedford, MA). A whole-body scan was taken of each participant while lying in the supine position. Body composition was calculated using the Hologic QDR scan software. The Hologic QDR 4500 is the same DXA chosen by the U.S. Centers for Disease Control and Prevention to measure body composition as part of NHANES (National Health and Nutrition Examination Survey) [17]. The Hologic DXA is considered a valid and reliable measurement method for the assessment of BF% [18–20]. To test concurrent validity, DXA results were compared to Bod Pod results for a subsample of 100 subjects from the current investigation. A Pearson correlation of 0.94 (*P* < 0.001) and an intraclass correlation of 0.97 (*P* < 0.001) were identified. Test-retest reliability of the Hologic DXA, with complete repositioning, when measured on the same subsample of 100 subjects from the present study, resulted in an intraclass correlation of 0.99 (*P* < 0.0001).

The 4500 W was calibrated at the start of each test day. To ensure accuracy, laser light cross-hairs produced by the 4500 W allowed the technician to reliably place participants in the same position on the table under the scanning arm.

#### 2.2.3. Waist Circumference

Abdominal obesity is a significant risk factor for a number of cardiovascular diseases. Careful measurement of the circumference of the abdomen at the umbilicus accurately accounts for differences in abdominal adiposity. According to Ross et al., waist circumference accounts for 91% of the variance in adipose tissue indexed using magnetic resonance imaging [21]. In the present study, waist circumference was measured using the protocol recommended by Lohman using a nonelastic measuring tape [22]. Subjects wore a standard university-issue swimsuit to minimize the effect of clothes. Two measurements were taken on each subject, and if the values were within 1 cm, the average was used. If the difference was greater than 1 cm, then a third measurement was taken and the average of the two closest values was used to index abdominal adiposity. A third measurement was needed for 18% of the participants. The test-retest means did not differ significantly and were less than 0.05 cm apart. Test-retest reliability for the waist circumference measurement using intraclass correlation was 0.99 (*P* < 0.0001).

### 2.3. Covariates

Physical activity, or lack of it, can influence body composition. Consequently, physical activity was assessed objectively using 7164 Actigraph accelerometers (ActiGraph, Pensacola, FL). Each participant received face-to-face training and written instructions about how to wear the device. Subjects were to wear the accelerometer on a standard, light-weight belt over the left hip. The accelerometer accurately assesses the intensity and duration of movement and can discriminate between sitting, light household activities, slow walking, vigorous walking, jogging, running, and other activities. More studies have employed Actigraph accelerometers to objectively measure physical activity outside the lab than any other device, and it has been validated many times [23, 24].

Participants wore the accelerometers continuously for 7 consecutive days, except while swimming or bathing. Participants were called twice during the week so questions could be answered and to encourage wear-time compliance. Data collected by the Actigraph were downloaded and evaluated for the non-wear time when returned at the end of the week. Average wear time from 7 : 00 am to 10 : 00 pm (15 hours) was 13.9 hours (93% wear-time compliance). Total physical activity was indexed objectively by adding all the raw activity counts over the 7 days. Many studies have used this procedure to index total physical activity objectively [25–27].

Energy intake has a significant effect on body composition. If measured accurately, as energy intake increases, body fat and waist circumference tend to increase, when physical activity is also taken into account. Consequently, energy intake was employed as a covariate in the present investigation.

Concurrent validity was shown for the energy intake variable used in the present study, with age and education being controlled. Specifically, calorie intake was a significant predictor of objectively measured physical activity (*F* = 7.3, *P*=0.0074), waist circumference (*F* = 14.5, *P*=0.0002), BMI (*F* = 15.6, *P* < 0.0001), bean intake (*F* = 5.3, *P*=0.0220), and the bean factor scores (*F* = 5.4, *P*=0.0214).

Age and education are both predictive of obesity. Older adults and those with less education tend to have more obesity than their counterparts. Hence, age and education level were included as covariates.

### 2.4. Statistical Analysis

Alpha was set at the 0.05 level and all *P* values were two-sided. SAS version 9.4 was used to conduct the statistical analyses (SAS Institute, Inc., Cary, NC, USA).

The magnitude of the linear relationship between bean consumption and body composition was evaluated using multiple regression and the General Linear Model procedure. Regression coefficients were used to quantify the extent of the linear relationships. Partial correlation was employed to adjust statistically for differences in potential mediating variables, including age, education, energy intake, and objectively measured physical activity.

Total bean and legume intake was measured using two different methods. First, serving size and frequency of the four bean-items of the Block Food Frequency Questionnaire were used to estimate cups of each bean dish consumed per year. Cups of the four bean food items were summed, generating a total cups of beans exposure variable. This value was converted to total cups of beans per 1000 kilocalories to negate the effect that subjects who eat the most calories tend to eat the most beans. The distribution of the bean intake variable deviated from normal, so it was log-transformed before inclusion in the analyses.

The second strategy to quantify bean and legume consumption was based on a principal component factor analysis employed to create a bean intake construct. A factor score was generated for each participant, representing adherence, or lack of adherence, to the bean construct or bean-based dietary pattern. Individual factor scores ranged from very strong adherence to very weak adherence, with a mean of 0.0 and a standard deviation of 1.0. Because some factor scores were negative, the factor scores could not be log-transformed, although they were not normally distributed. Therefore, the factor scores were categorized into tertiles, representing strong, moderate, and weak adherence to the bean intake dietary pattern. The extent to which mean levels of body fat percentage and waist circumference differed across the tertiles of bean intake adherence was determined using the analysis of variance (ANOVA). To assess the influence of differences in age, education, energy intake, and physical activity on the ANOVA results comparing tertiles, a partial correlation was employed. The least-squares means procedure was used to generate adjusted means based on the covariates.

Statistical power was calculated to determine the number of subjects needed to detect a correlation of 0.20 with four covariates controlled (i.e., age, education, energy intake, and physical activity), with alpha being fixed at 0.05 and power being set at 0.80. The SAS power calculation showed that 197 subjects were needed. Because the present investigation included 246 participants, statistical power was 0.88, given the parameters outlined.

## 3. Results

For the sample of 246 participants, the mean (±SD) bean and legume intake was 50.4 ± 49.7 cups per year. The mean bean intake per 1000 kcal per year was 25.6 ± 25.1 cups. The average body fat percentage was 32.5 ± 7.3 and the mean waist circumference was 82.7 ± 10.2 cm. The mean energy intake was 1973 ± 319 kilocalories (kcal) per day. On average, participants accumulated 2.63 ± 0.95 million activity counts across the week of physical activity monitoring. Cups of beans and legumes consumed were positively related to energy intake per day (*r* = 0.152, *P*=0.0170). However, when expressed as beans consumed per 1000 kcal, there was no association (*r* = −0.041, *P*=0.5206), and the relationship was weaker when the beans per 1000 kcal variable was log-transformed (*r* = −0.008, *P*=0.8975).  Table 1 shows the distribution, including the 5^th^, 25^th^, 50^th^, 75^th^, and 95^th^ percentiles, associated with the key exposure and outcome variables.

### 3.1. Factor Analysis Findings

The four bean food items loaded similarly on the bean factor, with a total eigenvalue of 1.8, accounting for 45% of the total variance. Specifically, refried beans or bean burritos had a factor loading of 0.63. The loading of the chili with beans was 0.71, that of baked beans, pintos, or other dried beans was 0.63, and that of the bean, split pea, or lentil soup was the highest at 0.72. The average bean factor score was 0.0 ± 1.0. Factor scores ranged from −1.0 to 5.7. Approximately 65% of the factor scores were negative.

### 3.2. Bean Intake and Body Fat Percentage

The association between bean and legume consumption per 1000 kcal and body fat percentage was linear, inverse, and significant. For each 10% increase in bean intake per 1000 kcal, body fat was 0.12 to 0.14 percentage point lower, on average, depending on the covariates included in the model. As shown in Table 2, adjusting for differences in the covariates had little effect on the regression results.

As displayed in Table 3, mean BF% levels differed significantly across the tertiles of bean and legume intake per 1000 kcal. The differences remained significant after adjusting for the covariates. With all the covariates controlled, BF% differed by almost 4 percentage points between women who were in the low bean intake tertile and those in the high intake tertile (*F* = 7.4, *P*=0.0008). After adjusting for all the covariates, the effect size between tertiles 1 and 3 was greater than 1/2 standard deviation.

Using factor scores to represent adherence to the bean and legume dietary pattern, differences in body fat percentages across the tertiles of adherence were statistically significant, as displayed in Table 4. After adjusting for differences in age, education, energy intake, and physical activity levels, mean body fat percentage differences remained significant (*F* = 7.9, *P*=0.0005).

### 3.3. Bean Intake and Waist Circumference

Waist circumference was correlated with BF% in the 246 women (*r* = 0.774, *P* < 0.0001). The relationship between bean consumption per 1000 kcal and waist circumference was linear and inverse, similar to the association between bean intake and body fat percentage. However, with waist circumference as the outcome variable, most of the associations were borderline significant. For each 10% increase in bean intake per 1000 kcal, waist circumference was 0.12 to 0.13 percentage point lower, on average (Table 2).

Table 3 shows that mean waist circumferences differed significantly across the tertiles representing low, moderate, and high bean and legume intake per 1000 kcal. Controlling for the covariates had little impact on the differences. After adjusting for age, education, energy intake, and physical activity level, the tertiles representing cups of bean consumption per 1000 kcal differed significantly in mean waist circumference (*F* = 4.2, *P*=0.0164).

Average waist circumferences also differed across tertiles based on factor scores, as revealed in Table 4. With all the covariates controlled, waist circumferences were significantly larger for those in the 1^st^ tertile, representing low adherence to the bean intake dietary pattern, compared to the other two tertiles (*F* = 4.5, *P*=0.0118).

## 4. Discussion

The present study was conducted to determine the association between legume intake, particularly bean consumption, and body fat percentage in women. Waist circumference was employed as a secondary outcome measure. An ancillary goal was to evaluate the effect of age, education, energy intake, and physical activity on the bean intake and body fat and waist circumference associations.

The key finding was that as bean and legume consumption increased, body composition tended to improve. The relationship for BF% was linear, inverse, and meaningful. Moreover, with participants being divided into tertiles, those who consumed low quantities of beans and legumes, or did not adhere to a bean-based dietary construct, had substantially higher levels of body fat and larger waist circumferences than their counterparts.

The regression analysis findings resulted in regression coefficients that were statistically significant, but not impressive on the surface (Table 2). For each 10% increase in bean consumption, BF% decreased by 0.12–0.14 percentage point. At first glance, 0.12–0.14 percentage point appears almost meaningless. However, it is important to note that there were substantial differences in bean and legume intakes among participants. As shown in Table 1, women at the 25^th^ percentile consumed only 10 cups of beans per 1000 kcal per year, whereas those at the 75^th^ percentile ate 33.7 cups per 1000 kcal per year, a difference of 237%. Although a 10% increase in bean consumption (i.e., from 10 cups to 11 cups per year) predicted little variation in body fat percentage, the 237% increase (i.e., from 10 cups to 33.7 cups) accounted for substantially lower levels of body fat.

Separating subjects into tertiles based on bean and legume intake provided results that were easy to interpret (Table 3). Comparing women in the lowest tertile to those in the highest tertile revealed a mean body fat difference of almost 4 percentage points and a difference of more than 4 cm in waist circumference, on average. Few dietary factors account for such large differences in body fat and abdominal obesity.

Most individual randomized controlled trials (RCTs) have failed to show that bean consumption contributes to weight and fat loss [2, 10]. However, when combined using meta-analysis, results have been promising [2, 10]. On the other hand, Williams indicates that there is not sufficient evidence to conclude that legumes help with weight management [12]. As mentioned previously, the problem may be that most individual RCTs have lacked statistical power, as sample sizes have been modest, at best. When statistical power is low, treatment effects must be large to be statistically significant. Moreover, RCTs require participants to adhere to an intervention, such as eating a significant quantity of beans. If subjects are not used to consuming beans, compliance could become a problem. When compliance is weak, small and insignificant treatment effects usually result.

In a cross-sectional study by Papanikolaou and Fulgoni [28], a 24-hour dietary recall was employed to identify bean consumers and nonconsumers. Bean consumers were found to have lower body weight and smaller waist circumferences than their counterparts. In short, like the present study, when bean consumption is evaluated, but compliance is not an issue, legume consumption is related to healthier body weights and waists.

Why would high bean and legume consumption predict favorable levels of body fat and waist circumference? What is the underlying mechanism? There are probably at least six contributing factors.

First, beans and legumes are low in dietary fat. Fat provides 9 kcal per gram, whereas carbohydrate and protein each afford 4 kcal per gram. Consequently, beans do not contain large amounts of food energy for their weight. They are not an energy-dense food. The literature shows clearly that low-fat foods are connected to reduced levels of body fat and obesity [29–33].

Second, legumes, including beans, are among the lowest glycemic index foods [34, 35]. In short, compared to other carbohydrate-rich foods, blood sugar levels increase less with the consumption of beans. They are often recommended to improve glycemic control [36, 37]. Several studies indicate that diets based on low glycemic foods, including beans, produce significant weight loss and reductions in abdominal obesity [11, 38, 39].

Third, beans are high in dietary fiber [40, 41]. One cup of beans contains approximately 10.4–15.6 grams of fiber [42]. Dietary fiber, particularly soluble fiber, can bind fats and sugars, decreasing their absorption and use by the body [43, 44]. Research shows unmistakably that adding dietary fiber to the diet reduces the risk of weight gain and fosters healthier body fat levels [45–48].

Fourth, in addition to dietary fiber, beans are a good source of plant protein. Consequently, beans and other legumes are considered both a vegetable food and protein food, according to the 2015–2020 US Dietary Guidelines [49]. No other food has this distinction. Protein is known to be the most satiating of the macronutrients, and many studies show that diets high in plant protein are predictive of decreased body weight [50–54].

Fifth, legumes have qualities that increase their capacity to satiate. Beans promote feelings of fullness and satisfaction, generally leading to reduced consumption of food and fewer calories [34]. Numerous investigations support the satiating power of legumes [55–57]. In a meta-analysis of 9 randomized cross-over trials, bean consumption was found to increase acute satiety by 31% compared to other foods [56]. Additionally, using a cross-over design, Reverri et al. showed that whole black beans increased cholecystokinin (CCK) and peptide tyrosine tyrosine (PYY), satiety-related GI hormones, compared to a meal with added fiber or a control meal [58].

Sixth, the gut microbiome is an immense ecosystem of microbes that affects the risk of several disorders. Studies indicate that beans and other legumes have a favorable impact on the intestinal microbiota of humans [59–61]. Bean consumption, leading to a healthier gut microbiome, could be another mechanism by which legumes help to improve body weight and adiposity [62–64].

So why might beans be a good choice in the battle against obesity? Beans are low in fat and the glycemic index. They are high in fiber and plant protein. They contribute significantly to satiety and they improve the gut microbiome. In short, beans possess a unique combination of dietary qualities. Each characteristic is individually associated with a decreased risk of obesity. However, beans are unusual because they possess all these qualities. It is likely because of these qualities that bean consumption accounted for lower levels of body fat and smaller waists in the present study.

The present study had limitations. First, it focused on 246 women, but no men. Consequently, the findings cannot be generalized to men. Second, because the study employed a cross-sectional design, cause-and-effect conclusions are not applicable. Bean intake could cause lower levels of body fat and smaller waists, but other unknown factors could explain the relationship. High bean intake could be a marker of a healthy lifestyle. Those who eat beans regularly might practice other behaviors that lead to improved weight management. Differences in physical activity are not likely the reason because activity was carefully monitored using accelerometers and controlled statistically. However, there are other potential mediating factors, such as diet, culture, and health consciousness, that could theoretically explain the inverse relationship detected between bean intake, BF%, and waist circumference.

The present study also had several strengths. The sample was relatively large and statistical power was very good. Additionally, a number of potential mediating factors were controlled statistically. Therefore, it is not likely that the relationship between bean intake and body composition was a function of differences in age, education, energy intake, or physical activity. Further, bean consumption was indexed using two methods: (1) the sum of bean-based food items quantified using serving size estimates and bean intake frequencies, and (2) factor scores estimating adherence to a bean-based dietary pattern. Lastly, two outcome measures, BF% and waist circumference, were used to quantify body composition.

## 5. Conclusion

In conclusion, beans are a unique food. They have multiple traits making them nutritious and filling. Beans are a vegetable and also a protein food. They are nutrient dense, but not energy dense. Although they have many favorable characteristics, consumption levels are low in most developed countries. In the present study, as bean intake increased and as adherence to a bean-based dietary pattern increased, body fat levels tended to decrease. Those with high intakes also had less abdominal obesity than their counterparts. It appears that beans and other legumes have dietary qualities that may be beneficial for weight management.

## Figures and Tables

**Table 1:** Percentiles for the exposure and outcome variables (*n* = 246).

Variable	Percentile				
	5^th^	25^th^	50^th^	75^th^	95^th^
Legumes (cups/yr)	6.0	20.5	35.0	60.0	139.0
Legumes (cups/1000 kcal/yr)	3.0	10.0	17.6	33.7	72.0
Bean construct (factor scores)	−0.9	−0.6	−0.3	0.3	1.7
Body fat percentage (%)	19.1	26.4	33.0	38.1	42.6
Waist circumference (cm)	69.0	74.3	80.9	91.0	99.8

Cups/yr: total cups of beans and legumes consumed per year. Cups/1000 kcal/yr: cups of beans and legumes consumed per 1000 kcal per year. Body fat percentage is expressed as a percentage × 100 . cm: centimeter.

**Table 2:** Magnitude of the linear associations between bean and legume intake, body fat percentage, and waist circumference in 246 women.

Body fat percentage				
Exposure variable (per 10% increase)	Regression coefficient	SE	*F*	*P*
Covariate				
Bean intake (cups/1000 kcal)				
None	−0.13	0.05	7.7	0.0058
Age	−0.14	0.05	8.2	0.0045
Age, energy intake	−0.14	0.05	8.2	0.0047
Age, PA	−0.13	0.05	7.8	0.0056
Age, PA, energy intake, education	−0.12	0.05	7.4	0.0069

Waist circumference (cm)				

Bean intake (cups/1000 kcal)				
None	−0.13	0.07	3.6	0.0576
Age	−0.13	0.07	3.8	0.0515
Age, energy intake	−0.13	0.07	3.9	0.0494
Age, PA	−0.12	0.07	3.5	0.0643
Age, PA, energy intake, education	−0.12	0.06	3.4	0.0680

SE: standard error of the regression coefficient. PA: physical activity. Interpretation of the regression coefficient results would be as follows for the model under body fat percentage with age, PA, energy intake, and education controlled: for each 10% increase in bean intake (cups per 1000 kcal), body fat tends to be 0.12 percentage points lower, on average.

**Table 3:** Mean differences in body fat percentage and waist circumference across tertiles of legume intake in women (*n* = 246).

	Bean intake tertiles		
Outcome	Low	Moderate	High	*F*	*P*
Covariate	Mean ± SD	Mean ± SD	Mean ± SD		
Body fat percentage					
None	34.7^a^±6.8	31.8^b^ ± 7.1	30.9^b^ ± 7.4	6.2	0.0023
Age	34.7^a^	31.7^b^	31.0^b^	6.4	0.0019
Age, energy intake	34.7^a^	31.7^b^	31.0^b^	6.3	0.0022
Age, PA	34.8^a^	31.7^b^	30.9^b^	7.7	0.0006
Age, PA, energy intake, educ	34.4^a^	31.2^b^	30.6^b^	7.4	0.0008

Waist circumference (cm)					
None	85.2^a^ ± 9.4	81.8^b^ ± 10.6	81.1^b^ ± 10.3	3.8	0.0230
Age	85.2^a^	81.8^b^	81.1^b^	3.9	0.0209
Age, energy intake	85.1^a^	81.8^b^	81.3^b^	3.6	0.0278
Age, PA	85.3^a^	81.7^b^	81.1^b^	4.3	0.0142
Age, PA, energy intake, educ	84.4^a^	80.8^b^	80.5^b^	4.2	0.0164

^a,b^Means on the same row with different superscript letters were significantly different (*P* < 0.05). SD = standard deviation. PA = physical activity. Educ = education level. Tertiles were based on cups of beans consumed per 1000 kcal. There were 82 subjects in each tertile. Means on the same row as the covariate(s) were adjusted for the covariate(s).

**Table 4:** Mean differences in body fat percentage and waist circumference across tertiles of bean and legume adherence, based on factor scores.

	Bean intake adherence		
Outcome	Low	Moderate	High	*F*	*P*
Covariate	Mean ± SD	Mean ± SD	Mean ± SD		
Body fat percentage					
None	34.9^a^ ± 6.4	31.5^b^ ± 7.2	31.0^b^ ± 7.6	7.5	0.0007
Age	35.0^a^	31.6^b^	31.0^b^	7.6	0.0006
Age, energy intake	35.0^a^	31.5^b^	30.9^b^	8.1	0.0004
Age, PA	34.8^a^	31.6^b^	31.0^b^	7.2	0.0009
Age, PA, energy intake, educ	34.5^a^	31.2^b^	30.6^b^	7.9	0.0005

Waist circumference (cm)					
None	85.1^a^ ± 9.4	81.7^b^ ± 10.7	81.4^b^ ± 10.2	3.3	0.0398
Age	85.1^a^	81.7^b^	81.4^b^	3.3	0.0383
Age, energy intake	85.5^a^	81.6^b^	81.1^b^	4.8	0.0094
Age, PA	85.3^a^	81.7^b^	81.1^b^	3.0	0.0536
Age, PA, energy intake, educ	84.6^a^	80.8^b^	80.5^b^	4.5	0.0118

^a,b^Means on the same row with different superscript letters were significantly different (*P* < 0.05). SD = standard deviation. PA = physical activity. Educ = education level. Categories were tertiles based on adherence to the dietary pattern associated with bean and legume consumption. The sample sizes were Tertile 1 (*n* = 81), Tertile 2 (*n* = 83), and Tertile 3 (*n* = 82). Means on the same row as the covariate(s) were adjusted for the covariate(s).

## Data Availability

The present study data are available from the corresponding author upon request.
